# Gesundheitsprobleme nach radikaler Prostatektomie: Welche subjektiven Gesundheitseinschränkungen sind für Langzeitüberlebende relevant?

**DOI:** 10.1007/s00120-024-02441-0

**Published:** 2024-09-20

**Authors:** Tobiasz Klorek, Anton N. J. H. Schlichte, Cornelia Peter, Matthias Jahnen, Andreas Dinkel, Stefan Schiele, Lukas Lunger, Helga Schulwitz, Jürgen E. Gschwend, Kathleen Herkommer

**Affiliations:** 1 Klinik und Poliklinik für Urologie, Technische Universität München, School of Medicine, TUM Universitätsklinikum rechts der Isar, Ismaninger Straße 22, 81675 München, Deutschland; 2 Klinik und Poliklinik für Psychosomatische Medizin und Psychotherapie, Technische Universität München, School of Medicine, TUM Universitätsklinikum rechts der Isar, München, Deutschland

**Keywords:** Self-Administered Comorbidity Questionnaire, Subjektive Komorbiditäten, Prostatakarzinom-Langzeitüberlebende, Beeinträchtigung, „Patient-reported outcomes“, “Self-Administered Comorbidity Questionnaire”, Subjective health restrictions, Prostate cancer survivors, Impairment, Patient-reported outcomes

## Abstract

**Hintergrund:**

Die radikale Prostatektomie (RP) ist eine der häufigsten Therapiestrategien zur Behandlung des lokal begrenzten Prostatakarzinoms (PCa). Derzeit ist nicht klar, welchen Stellenwert postoperative funktionelle Einschränkungen im Langzeitverlauf für betroffene Patienten haben, insbesondere im Vergleich zu altersbedingten Komorbiditäten.

**Ziel der Arbeit:**

Ziel dieser Analyse war es, die Prävalenz subjektiver Gesundheitsprobleme sowie funktioneller Defizite bei PCa-Langzeitüberlebenden nach RP und die erlebte Beeinträchtigung zu quantifizieren.

**Material und Methoden:**

Mittels des deutschsprachigen „Self-administered Comorbidity Questionnaire“ (SCQ-D) bewerteten 3173 Langzeitüberlebende nach RP ihre Begleiterkrankungen in 13 vorgegebenen Kategorien sowie in 3 Freitextantwortfeldern. Die Beurteilung erfolgte anhand der Dimensionen „Problem“, „Behandlung“ und „Beeinträchtigung“.

**Ergebnisse:**

Das Alter bei Befragung betrug im Mittel 79,5 (SD ± 6,4) Jahre, die Zeit seit RP 17,4 (SD ± 3,7) Jahre. Die drei am häufigsten als Problem angegebenen Komorbiditäten/der Anteil der Patienten, die sich beeinträchtigt fühlten waren: Bluthochdruck (62,2 %/8,5 %), Rückenschmerzen (44,1 %/54,5 %) und Arthrose (36,1 %/54,1 %). Am häufigsten waren unter dem Überbegriff der „urologischen Probleme“ (6,1 %/72,7 %): Inkontinenz (4,8 %/74,3 %), Blasenprobleme (1,1 %/61,8 %), erektile Dysfunktion (0,5 %/47,1 %).

**Schlussfolgerung:**

Insgesamt wurden nicht-karzinombedingte Komorbiditäten im Langzeitverlauf nach RP zwar häufig als „Problem“ wahrgenommen, sie sind aber selten mit einer erlebten Beeinträchtigung verknüpft. Demgegenüber wurden tumortherapiebedingte, urologische Probleme selten als „Problem“ angegeben, sie werden allerdings sehr häufig als beeinträchtigend im Alltag erlebt.

## Abstract (en)

**Background:**

Radical prostatectomy (RP) is one of the most common therapeutic strategies for treating localized prostate cancer (PCa). Currently, the significance of postoperative functional limitations for affected patients in the long-term course, especially in comparison to age-related comorbidities, is unclear.

**Objective:**

The aim of this study was to quantify the prevalence of subjective health restrictions alongside functional deficits in long-term PCa survivors after RP and their relevance for subjective impairments in everyday life.

**Materials and methods:**

Using the German version of the Self-Administered Comorbidity Questionnaire (SCQ-D), 3173 long-term survivors after RP reported their comorbidities in 13 predefined categories and in 3 free-text fields along the dimensions “problem,” “treatment,” and “impairment”.

**Results:**

The mean age at survey was 79.5 years (standard deviation, SD ± 6.4), with a mean time since RP of 17.4 years (SD ± 3.7). The three most frequently identified comorbidities/percentage of patients who felt impaired were: hypertension (62.2%/8.5%), back pain (44.1%/54.5%), and osteoarthritis (36.1%/54.1%). The most frequently mentioned additional health problems can be subsumed under the umbrella term “urological problems” (6.1%/72.7%): incontinence (4.8%/74.3%), bladder problems (1.1%/61.8%), and erectile dysfunction (0.5%/47.1%).

**Conclusion:**

In summary, non-cancer-related comorbidities in the long-term course after RP are often perceived as “problems” but rarely lead to subjective impairment. In contrast, treatment-related urological problems are rarely reported as “problems”, but they very often lead to subjective impairment in everyday life.

## Einleitung

Fortschritte in der Diagnostik und Therapie haben in den letzten Jahrzehnten zu einer wachsenden Anzahl von Langzeitüberlebenden nach Primärtherapie des lokalisierten Prostatakarzinoms (PCa) geführt [1]. Mit zunehmendem Alter der Langzeitüberlebenden wächst aber auch die Rate altersbedingter Komorbiditäten: Eine retrospektive Studie an Hausarztpraxen in Deutschland konnte zeigen, dass rund 25 % einer repräsentativen Kohorte von 840.000 Menschen über 65 Jahre an mindestens 4 Komorbiditäten litten [2]. Die Berücksichtigung dieser Aspekte im Rahmen der Therapieentscheidung ist essentiell, insbesondere da die Art der Primärtherapie unterschiedliche Nebenwirkungen und möglicherweise chronische, funktionelle Einschränkungen mit sich bringen kann (z. B. persistierende Inkontinenz oder erektile Dysfunktion; [1, 3–5]).

Es liegt nahe, dass sowohl Komorbiditäten als auch funktionelle Einschränkungen nach einer radikalen Prostatektomie (RP) einen deutlichen Leidensdruck verursachen und sich negativ auf die Lebensqualität der Betroffenen auswirken können. Derzeit ist in der Literatur allerdings nicht ausreichend geklärt, ob und wie stark mit der Primärtherapie assoziierte funktionelle Einschränkungen im Langzeitverlauf im Alltag beeinträchtigend und relevant für Langzeitüberlebende nach der Primärtherapie des lokalisierten PCa sind, insbesondere im Vergleich zu nicht-krebsassoziierten Komorbiditäten. Die Prostatakrebsbehandlung erfordert eine ganzheitliche Betrachtung der Erkrankung und sollte sich daher nicht nur auf krebsbezogene Faktoren hinsichtlich Krebsprogression und Überleben beschränken. Ein besseres Verständnis hinsichtlich Prävalenz und subjektiver Beeinträchtigung durch vorliegende Komorbiditäten bzw. funktionelle Einschränkungen nach RP kann helfen, auf die Bedürfnisse dieser spezifischen Patientengruppe während der Nachsorge einzugehen.

Das Ziel dieser Fragebogenstudie war es daher, die Prävalenz von subjektiven Gesundheitsproblemen neben funktionellen Einschränkungen bei PCa-Langzeitüberlebenden nach RP und deren Beeinträchtigung für die betroffenen Patienten zu quantifizieren.

## Material und Methoden

Im Rahmen der jährlichen Nachsorge wurden PCa-Langzeitüberlebende nach radikaler Prostatovesikulektomie des nationalen Forschungsprojekts „Familiäres Prostatakarzinom“ im Herbst 2021 postalisch mittels Fragebögen kontaktiert. Der Fokus der Befragung lag auf den subjektiven Komorbiditäten, welche mittels der deutschsprachigen Version des SCQ (Self-Administered Comorbidity Questionnaire; SCQ-D) erfasst wurden [6]. Der SCQ‑D ist ein Selbsteinschätzungsinstrument für Patienten und enthält 13 vorgegebene Gesundheitsprobleme sowie 3 Freitextantwortfelder für zusätzliche Gesundheitsprobleme, welche jeweils in Bezug auf „Problem“, „Behandlung“ und „Beeinträchtigung“ mit ja/nein beantwortet werden können. Für die Analyse lag der Fokus auf den Skalen „Problem“ und „Beeinträchtigung“, die Skala „Behandlung“ wird nur deskriptiv in Tab. 3 dargestellt. Die von den Patienten in den Freitextantworten angegebenen Gesundheitsprobleme wurden unabhängig von 2 Ärzten in 14 weitere Zusatzkategorien eingeteilt.

Zusätzlich wurden soziodemografische, klinische und psychosoziale Parameter erhoben. Unter anderem wurden Depression und Angst mithilfe des Patient Health Questionnaire‑4 (PHQ-4) erfasst, einem Screening-Instrument mit 4 Items, das aus einer Depressionsskala (PHQ-2) und einer Angstskala (GAD-2) besteht [7]. Ein Punktwert von ≥ 3 auf der jeweiligen Subskala deutet auf klinisch relevante Symptome für Depression und Angst hin. „Frailty“ wurde mithilfe des Groningen Frailty Index (GFI) erhoben, wobei Patienten mit einem Score ≥ 4 als „frail“ eingestuft wurden [8]. Die Lebensqualität wurde mittels des EORTC Quality of Life Questionnaire (QLQ-C30; Item 29 und 30) erhoben [9].

Alle Parameter wurden entweder als Mittelwerte und Standardabweichungen (SD), Mediane und Interquartilbereiche (IQR) oder prozentuale Anteile (%) angegeben. Für diese Studie wurden nur deskriptive Statistiken durchgeführt. Ein positives Ethikvotum der Ethikkommission des Klinikums rechts der Isar liegt vor.

## Ergebnisse

Insgesamt wurden 5652 Langzeitüberlebende nach RP kontaktiert, von 3173 Patienten konnten vollständig ausgefüllte Fragebögen in die Auswertung einfließen, was einer Rücklaufquote von 56,1 % entspricht.

Eine Beschreibung der Studienkohorte ist in Tab. 1 dargestellt. Das durchschnittliche Alter bei Befragung betrug 79,5 ± 6,4 Jahre, die vergangene Zeit seit RP 17,4 ± 3,7 Jahre. Unter den Langzeitüberlebenden befanden sich 10,4 % zum Zeitpunkt der Befragung unter Therapie, davon 99,4 % unter Androgendeprivation. Weniger als die Hälfte der Patienten hatte eine hohe Schulbildung (43,7 %), der Großteil lebte in einer Partnerschaft (86,5 %), hatte Kinder (88,5 %) und war mit seiner wirtschaftlichen Situation mindestens zufrieden (98,1 %; s. Tab. 1).

**Tab. 1:** Soziodemografische und klinische Merkmale des Studienkollektivs (*n* = 3173)

Soziodemografische und klinische Merkmale	%	*n*
**Alter bei Befragung, Jahre, Mittelwert (±** **SD)**	79,5 (± 6,4)	
**Zeit seit RP, Jahre, Mittelwert (±** **SD)**	17,4 (± 3,7)	
**Anzahl Gesundheitsprobleme, Mittelwert (±** **SD)**	3,0 (± 1,9)	
**Aktuelle Therapie**		
*Nein*	89,6	2844
*Ja*	10,4	329
Androgenentzug	99,4	327
Chemotherapie	0,3	1
Bestrahlung	0,3	1
**Bildungsniveau**		
*Niedrig*	39,2	1190
*Mittel*	17,1	519
*Hoch*	43,7	1326
**Aktuelle Partnerschaft**		
*Ja*	86,5	2695
*Nein*	13,5	419
**Kinder**		
*Ja*	88,5	2766
*Nein*	11,5	360
**Wirtschaftliche Situation**		
*Sehr gut oder gut*	72,2	2261
*Zufriedenstellend*	25,9	809
*Weniger gut oder schlecht*	1,9	60

*SD* Standardabweichung, *RP* radikale Prostatektomie

Die Lebensqualität lag im Mittel bei 66,0 ± 19,4. Bei insgesamt 10,9 % zeigte sich ein positives Screening für Depression (PHQ-2 ≥ 3 Punkte) und bei 8,4 % ein positives Screening für Angst (GAD-2 ≥ 3 Punkte). Als „frail“ eingestuft wurden 33,0 % der Patienten (GFI ≥ 4 Punkte; s. Tab. 2).

**Tab. 2:** Psychosoziale Merkmale des Studienkollektivs (*n* = 3173)

Psychosoziale Merkmale	%	*n*
*Screening auf Depression (PHQ-2)*		
Positiv (≥ 3)	10,9	331
Negativ (< 3)	89,1	2716
*Screening auf Angst (GAD-2)*		
Positiv (≥ 3)	8,4	253
Negativ (< 3)	91,7	2777
*Lebensqualität, Mittelwert (±SD)*	66,0 (± 19,4)	
*Lebensqualität, Median (IQR)*	66,7 (50,0–83,3)	
*Screening auf Frailty (GFI)*		
Positiv (≥ 4)	33,0	979
Negativ (≤ 3)	67,0	1985

*PHQ* Patient Health Questionnaire, *GAD* „generalized anxiety disorder“, *GFI* Groningen Frailty Index, *IQR* Interquartilsabstand

**Abb. 1:**
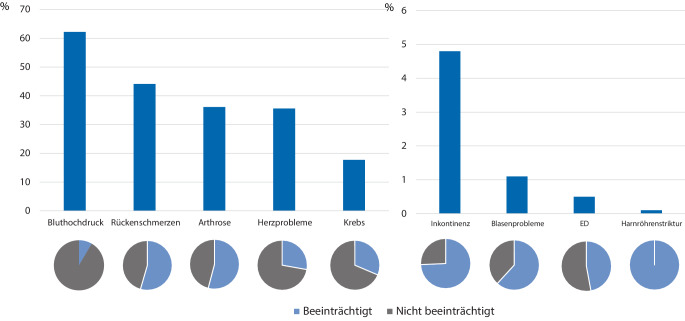
Die 5 häufigsten nicht-urologischen sowie die 3 häufigsten urologischen Gesundheitsprobleme (Anteil der Patienten mit Angabe als „Problem“ in %)

Im Mittel gaben die Patienten 3,0 (± 1,9) Gesundheitsprobleme an. Die am häufigsten als Problem angegebene Komorbidität war Bluthochdruck (62,2 %), durch den sich 8,5 % der Patienten subjektiv beeinträchtigt fühlten. Gefolgt von Rückenschmerzen (von 44,1 % der Patienten angegeben/54,5 % fühlten sich subjektiv beeinträchtigt), Arthrose (36,1 %/54,1 %), Herzproblemen (35,6 %/27,8 %) und Krebs (17,7 %/31,4 %; s. Tab. 3; Abb. 1 und 2). Die 3 am häufigsten genannten Zusatzkategorien in den Freitextantworten waren: urologische Probleme (6,1 %/72,7 %), neurologische Probleme (5,3 %/79,0 %) und Bewegungsapparatprobleme (4,8 %/71,7 %). Unter dem Überbegriff „urologische Problem“ wurden die Probleme Inkontinenz (4,8 %/74,3 %), Blasenprobleme wie „Reizblase“ oder „starker Harndrang“ (1,1 %/61,8 %), erektile Dysfunktion (0,5 %/47,1 %) und Harnröhrenstrikturen (0,1 %/100 %) zusammengefasst (s. Tab. 3).

**Tab. 3:** Subjektive Komorbiditäten (als „Problem“, „Behandlung“, „Beeinträchtigung“ angegeben) in absteigender Häufigkeit

Gesundheitsprobleme	„Problem“		„Behandlung“		„Beeinträchtigung“	
	%	*n*	%	*n*	%	*n*
Bluthochdruck^a^	62,2	1.973	94,6	1.867	8,5	167
Rückenschmerzen^a^	44,1	1.400	38,2	535	54,5	763
Arthrose^a^	36,1	1.145	36,2	415	54,1	619
Herzprobleme^a^	35,6	1.131	93,1	1.053	27,8	314
Krebs^a^	17,7	563	73,2	412	31,4	177
Diabetes/Blutzucker^a^	16,8	534	83,7	447	17,6	94
Magen-Darm-Probleme^a^	16,5	522	59,0	308	37,9	198
Lungenprobleme^a^	10,8	342	67,5	231	49,7	170
Nierenprobleme^a^	7,8	248	69,8	173	24,6	61
Depression^a^	7,3	231	49,8	115	45,9	106
Anämie oder andere Blutprobleme^a^	6,6	210	75,7	159	20,0	42
Rheuma^a^	6,6	209	52,2	109	52,6	110
Neurologische Probleme	5,3	167	70,1	117	79,0	132
Bewegungsapparatprobleme	4,8	152	54,6	83	71,7	109
*Inkontinenz*	*4,8*	*152*	*40,1*	*61*	*74,3*	*113*
Sonstiges	2,6	82	52,4	43	63,4	52
HNO-Probleme	2,1	65	47,7	31	64,6	42
Leberprobleme^a^	1,9	61	37,7	23	8,2	5
Augenprobleme	1,7	54	83,3	45	59,3	32
Hormon- und Stoffwechselprobleme	1,1	35	82,9	29	17,1	6
*Blasenprobleme*	*1,1*	*34*	*35,3*	*12*	*61,8*	*21*
Hautprobleme	1,0	31	80,7	25	45,2	14
Gefäßprobleme	0,7	21	81,0	17	42,9	9
Schlafprobleme	0,6	19	42,1	8	47,4	9
*Erektile Dysfunktion*	*0,5*	*17*	*29,4*	*5*	*47,1*	*8*
Psychische Probleme	0,1	3	0	0	33,3	1
*Harnröhrenstriktur*	*0,1*	*3*	*66,7*	*2*	*100*	*3*

Urologische Kategorien *kursiv* hervorgehoben

^a^Originalkategorien des SCQ‑D

**Abb. 2:**
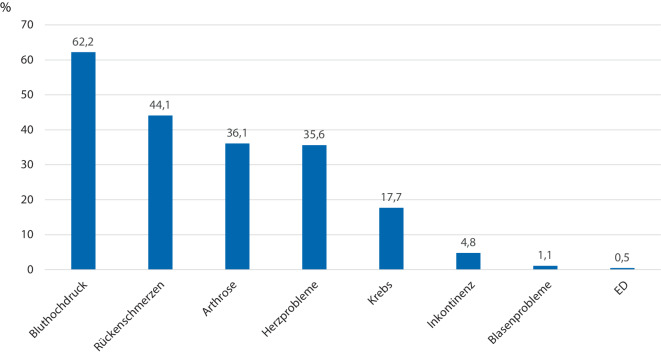
Die 5 häufigsten nicht-urologischen sowie die 4 urologischen Gesundheitsprobleme (Anteil der Patienten mit Angabe als „Problem“ in %). Darunter angegeben der Anteil, der das jeweilige Gesundheitsproblem als „Beeinträchtigung“ empfindet

## Diskussion

Für diese Studie wurden knapp 3200 Fragebögen ausgewertet. Im Schnitt gaben 80-jährige Langzeitüberlebende mehr als 17 Jahre nach RP an, an drei subjektiven Gesundheitsproblemen zu leiden. Am häufigsten wurden Bluthochdruck, Rückenschmerzen, Arthrose und Herzprobleme genannt. Diese Beschwerden spiegeln typische Folgeerscheinungen von Alter und Lebensstil wider, die viele Menschen in dieser Lebensphase beschreiben [2].

### Leise Beschwerden, lauter Einfluss: urologische und nicht-urologische Herausforderungen nach Prostataentfernung

Wider Erwarten wurden urologische Beschwerden im Langzeitverlauf nach RP sehr selten von den Patienten als „Problem“ angegeben (von nur rund 6 % der Befragten genannt). Andererseits stellen diese subjektiven, urologischen Gesundheitsprobleme eine erhebliche Beeinträchtigung für die wenigen Betroffenen dar (beispielsweise gaben nur 4,8 % der Teilnehmer an, an einer Inkontinenz zu leiden; davon fühlten sich jedoch 74,3 % der Betroffenen im Alltag beeinträchtigt). Andere Kollektive zeigten im Langzeitverlauf 6–8 Jahre nach offen-retropubischer/roboterassistierter RP aus funktioneller Sicht je nach Evaluationsmethode Inkontinenzraten von bis zu 29 % und einen Anteil von Patienten mit ED von 60–70 % [10, 11]. Nicht-urologische Gesundheitsprobleme wurden in unserem Kollektiv im Vergleich dazu deutlich häufiger angegeben. Interessanterweise fühlten sich aber im Verhältnis viel weniger Befragte durch diese beeinträchtigt (z. B. wurde arterielle Hypertonie von 62,2 % der Patienten als „Problem“ klassifiziert, nur 8,5 % fühlten sich durch diese beeinträchtigt). Diese unerwartete Diskrepanz zwischen der objektiven Erwartung von Häufigkeiten der Gesundheitseinschränkungen durch die RP und der tatsächlichen, subjektiven Wahrnehmung kann einerseits an dem hohen durchschnittlichen Alter der befragten Patienten liegen. Möglicherweise sehen 80-Jährige nach RP ihre Gesundheitsprobleme nicht als „Problem“ an, da sie bereits an das Vorliegen funktioneller Einschränkungen adaptiert sind. Urologische Probleme werden beispielsweise von den Patienten möglicherweise als gewöhnliche Alterserscheinung wahrgenommen, nicht (mehr) als Folge der onkologischen Therapie. Nur 17,7 % der Patienten gaben „Krebs als Problem“ an und nur 31,4 % erlebten dieses als beeinträchtigend. Weiterhin könnte der sehr niedrige Anteil der Patienten, die beispielsweise Inkontinenz oder erektile Dysfunktion als „Problem“ angaben, durch das Fehlen der entsprechenden Kategorien im SCQ‑D bedingt sein. Würden urologische Gesundheitseinschränkungen explizit in vorgegebenen Kategorien abgefragt, wäre möglichweise eine deutlich höhere Prävalenz zu erwarten. Jedoch fühlen sich Patienten durch urologische Gesundheitseinschränkungen sehr stark beeinträchtigt. Dieses scheinbare Paradoxon unterstreicht die Notwendigkeit, seltene, möglicherweise im Rahmen einer Adaptation vernachlässigte oder durch die Betroffenen tabuisierte Themen wie Inkontinenz oder erektile Dysfunktion insbesondere im Rahmen der urologischen Langzeitnachsorge gezielt anzusprechen. Im untersuchten Studienkollektiv fand sich auch bei 10,9 und 8,4 % der Teilnehmer ein positives Screening auf Depression und Angst – mehr als doppelt so häufig als im Vergleich zur Normalbevölkerung (5,9 % bezüglich Depressivität und 2,4 % bezüglich Angst [12]). Eine fundierte Aufklärung und einfühlsame Kommunikation zwischen Arzt und Patient ist unerlässlich, um auch diese Probleme anzusprechen und angemessene Unterstützung und Therapieoptionen anzubieten.

### Einfache Lösung, große Wirkung: auf dem Weg zu einer ganzheitlichen Nachsorge mit dem SCQ

Wie beispielsweise anhand von Patientenkollektiven mit Gon- und Koxarthrose oder terminaler Niereninsuffizienz bereits gezeigt wurde, kann der SCQ‑D einfach und ressourcenschonend angewendet werden [6, 13], eine komplizierte statistische Auswertung ist nicht notwendig. Die Beantwortung des Fragebogens konnte in vorliegender Studie durch 99 % der im Schnitt 80-jährigen Patienten komplett durchgeführt werden. Die Verwendung des SCQ‑D als Fragebogen könnte künftig auch im urologischen Praxisalltag hilfreich sein, die Hemmschwelle zur Angabe vorliegender Beschwerden/Komorbiditäten zu senken und besonders stark belastete Betroffene im Rahmen der Nachsorge gezielt zu identifizieren. Als positiver Nebeneffekt wird die Einbindung der Patientenperspektive in den Nachsorgeprozess im Sinne einer ganzheitlichen, patientenzentrierten Behandlung gefördert. Besonders informativ war die Analyse der Freitextfelder in dieser Auswertung, sie weist auf einen kritischen Mangel des Fragenbogens im „Standardformat“ hin: Die alleinige Verwendung der 13 vorgegebenen SCQ-D-Kategorien ohne Beachtung der Freitextantwortfelder hätte zu einem erheblichen Informationsverlust geführt, nicht nur in Hinblick auf urologische Gesundheitseinschränkungen. Beispielsweise waren ophtalomologische oder HNO-assoziierte Komorbiditäten in diesem Kollektiv selten (je ≤ 2,1 %), tatsächlich fühlte sich aber ein Großteil der Patienten (> 59 %) durch diese beeinträchtigt. Auch in anderen Kollektiven konnte gezeigt werden, dass die in den Freitextantwortfeldern angegebenen Gesundheitsprobleme eine hohe Relevanz für die Gesamtbetrachtung des Patientenwohlbefindens haben. Beispielsweise gaben insgesamt 43,9 % der befragten Patienten mit ankylosierender Spondylitis mindestens ein Gesundheitsproblem in den Freitextfeldern an, darunter insbesondere chronisch-entzündliche Darmerkrankungen, Uveitis und Psoriasis [14]. Auch im Rahmen der Entwicklung und Validierung des SCQ‑D wurden zusätzlich zu den vorgegebenen Kategorien von 20 % der Befragten Gesundheitsprobleme in die Freitextantwortfelder eingetragen, darunter am häufigsten Hyperthyreoidismus, periphere arterielle Verschlusskrankheit und benignes Prostatasyndrom [15]. Für eine breitere Anwendung des SCQ‑D bei älteren urologischen Patienten könnte daher die Erweiterung des Fragebogens um spezifische urologische Beschwerden wie Inkontinenz, erektile Dysfunktion oder Blasenprobleme bei den vorgegebenen Kategorien sinnvoll sein. Inwiefern der Fragebogen um zusätzliche, nicht-urologische Kategorien erweitert werden sollte, könnte durch weitere Analysen untersucht werden.

### Patientenzentriertes Arbeiten auf einen Blick: gezielte Befragung mit wenig Zeitaufwand

„Patient-reported outcomes“ gewinnen in allen Fachdisziplinen immer mehr an Bedeutung. Die subjektive Wahrnehmung von allgemeinen und fachspezifischen Gesundheitseinschränkungen hat nicht nur Auswirkungen auf die Primärtherapie, sondern v. a. auf die Rekonvaleszenz und das langfristige gesundheitliche Outcome. Eine besondere Herausforderung im Rahmen einer onkologischen Langzeitnachsorge liegt in der Identifizierung und adäquaten Versorgung von Patienten, die sich durch funktionelle Einschränkungen besonders beeinträchtigt fühlen. Häufig fehlen jedoch im Klinik- und Praxisalltag Mittel und Zeit, auf alle Bedürfnisse dieser besonderen Patientengruppe einzugehen. Prospektive Nachsorgestudien wie die PRO-P-Studie und der Einsatz des modifizierten SCQ-D-Fragebogens könnten die Versorgung von Patienten nach RP verbessern. Unsere Studie zeigte eine geringe Prävalenz urologischer Probleme im Langzeitverlauf – es ist jedoch zu beachten, dass diese Prävalenz aufgrund der Verwendung von Freitextfeldern zur Beantwortung von Fragen zu urologischen Problemen möglicherweise unterschätzt wurde. Der Einsatz von standardisierten Fragebögen wie dem urologisch-modifizierten SCQ‑D könnte daher dazu beitragen, die Prävalenz und Belastung durch urologische Probleme besser einschätzen zu können, z. B. ohne großen Aufwand während der Wartezeit in der Praxis. So könnten subjektiv belastende urologische Gesundheitseinschränkungen bei Langzeitüberlebenden nach RP patientenzentriert in die Nachsorgeroutine integriert, identifiziert und gezielt angesprochen werden. Darauf aufbauend kann mittels adäquater Versorgung einer möglichen Einschränkung im sozialen Leben vorgebeugt werden. Des Weiteren können vorliegende Ergebnisse auch im präoperativen Setting Anwendung finden. Unsere Studie zeigt, dass bestimmte urologische Beschwerden, wenngleich selten, eine erhebliche Belastung für Patienten darstellen können. Diese Erkenntnisse können dazu beitragen, das präoperative Aufklärungsgespräch zu verbessern, indem sie helfen, Patienten ein klareres Bild von den potenziellen Langzeitfolgen und deren Belastung nach RP zu vermitteln. Letztlich ist das Treffen einer informierten Entscheidung ein essentielles Ziel eines erfolgreichen Arzt-Patienten-Gesprächs und fußt auf der für Patienten adäquaten Aufbereitung medizinisch relevanter Informationen [16]. Insbesondere in der Ära des „shared decision making“, der gemeinsamen Entscheidungsfindung, kann ein besseres Verständnis über die mögliche Belastung durch Langzeitkomplikationen unter Langzeitüberlebenden nach RP behandelnden Ärzten helfen, eine umfänglichere Beratung hinsichtlich der Wahl der Primärtherapie anzubieten.

## Fazit für die Praxis

- Zusammengefasst unterstreichen die Ergebnisse dieser Studie die Notwendigkeit eines ganzheitlichen, patientenzentrierten Ansatzes zur Betreuung von Langzeitüberlebenden nach radikaler Prostatektomie (RP).
- Die Vielschichtigkeit der allgemeinen und urologischen Gesundheitseinschränkungen, die physischen und psychosozialen Herausforderungen sowie die Unterschiede in der objektiven Erwartung und subjektiven Wahrnehmung des Wohlbefindens betonen die Komplexität der Betreuung Prostatakarzinom (PCa)-Langzeitüberlebender.
- Die Erkenntnisse aus dieser Studie liefern wertvolle Informationen für die klinische Praxis, indem sie die Bedeutung einer individualisierten und umfassenden Versorgung betonen.
- Die Identifizierung und optimale Betreuung von subjektiv stark beeinträchtigten Patienten stellt in der Arbeitsroutine eine große Herausforderung dar, die unter Zuhilfenahme von Screening-Fragebögen wie einem urologisch modifizierten „Self-administered Comorbidity Questionnaire“ (SCQ-D) deutlich erleichtert werden könnte.

## Data Availability

Die erhobenen Datensätze können auf begründete Anfrage in anonymisierter Form beim korrespondierenden Autor angefordert werden. Die Daten befinden sich auf einem Datenspeicher am TUM Universitätsklinikum rechts der Isar München.
